# Supplementary material for: What drives the effectiveness of social distancing in combating COVID-19 across U.S. states?
Source: PLoS One. 2025 May 12;20(5):e0308244. doi: 10.1371/journal.pone.0308244 (PMC12068638; doi:10.1371/journal.pone.0308244)
Supplement: Attachment — Submitted filename: Response to editor.pdf [file pone.0308244.s001.pdf]

Dear Editor,

Thank you very much for the opportunity to revise our work, for potential publication in PLOS One. We have followed your advice closely and have reworked the introduction and discussion section of the paper to more clearly highlight the contribution of our work to the literature.

We believe that this has helped to strengthen the paper considerably, and are indebted to you for your guidance. Please see below a detailed response to your previous review. We have reprinted your comments in bold and are responding in normal font.

I enjoyed reading this manuscript, and believe that it holds promise. At the same time, I identified several interconnected issues that require the authors' attention before the manuscript can be considered for publication. First, the main argument needs to be worked out and formulated much more clearly. The main argument should be included in the paper's introduction.

Thank you for this excellent suggestion. We now highlight our main contribution already in the abstract, which we reprint here for your convenience:

"We propose a new theory of information-based voluntary social distancing in which people's responses to disease prevalence depend on the credibility of reported cases and fatalities and vary locally. We embed this theory into a new pandemic prediction and policy analysis framework that blends compartmental epidemiological/economic models with Machine Learning. We find that lockdown effectiveness varies widely across US States during the early phases of the COVID-19 pandemic. We find there would have been 240,000 more fatalities by June 2020 if people in all states would have voluntarily socially distanced like people in the least informed US state, and 25,000 fewer fatalities if people in all states would have responded like people in the most informed state."

Additionally, we extensively discuss our main contribution in the introduction (see below).

Second, a clear argument will make it easier for the authors to highlight their main contribution to the existing academic literature, which is relatively vague right now.

Thank you for this suggestion. We have followed your advice and completely rewritten the introduction of the paper to more clearly emphasize the contribution to the academic literature. We reprint the key passages discussing our contribution to the literature in the following:

"This paper complements the existing literature through three distinct contributions. Our first contribution is a new theoretical model of information-based voluntary social distancing, in which people learn about infection risks from publicly available data on new cases and fatalities. Importantly, the strength of voluntary social distancing responses to reported local cases and fatalities is driven by the perceived credibility of this data, which can be influenced by local governments' information policies and which will vary locally. In this context, our model predicts that local information policies that produce less credible case and fatality counts lead to a lower signal-to-noise ratio of published case and fatality counts. As a consequence, local information

policies will impact the degree of voluntary social distancing directly and the effectiveness of lockdown policies indirectly. This insight goes well beyond the analysis of [7] and has important policy implications. We are not aware of any other paper in the literature providing this insight or any similar policy analysis.

Our second contribution is methodological, in that we propose a new prediction and policy analysis framework combining an extended compartmental epidemiological model with Machine Learning. We embed our theory of information-based social distancing into a rich compartmental model including a variety of often unobserved, time-varying factors such as asymptomatic transmission, symptom-based testing and quarantining, and time-variation in fatality rates. In addition to daily local data on new COVID-19 cases and fatalities, we calibrate the model to local, daily cellphone-based data on mobility, which we establish as valid proxy for economic activity. Our framework can therefore provide cost-benefit analysis for policy makers interested in the economic and health effects lockdown policies. To increase the reliability of model forecasts, we use ensemble learning and cross-validation, two ideas from Machine Learning. Specifically, we re-calibrate the model for different time horizons and then use a weighted average of these estimates, to increase the robustness of out-of-sample predictions. The ensemble weights are then chosen via cross-validation, which minimizes the out-of-sample prediction error. Using these additional steps allows us to generate more generalizable patterns that are less likely driven by statistically noisy initial conditions. Of the 136 COVID-19 forecasting models reviewed in [1], only 3 papers use a hybrid approach between Machine Learning and compartmental models in combination with mobility data. All of these papers use mobility data as a predictor for new cases and fatalities but none of these three papers allows for a feedback of case and fatality data to mobility, the way our paper does. This feedback is a direct consequence of our theory of information-based voluntary social distancing and is therefore a main focus of our analysis.

Our third contribution consists of novel empirical results from the application of our new model and methodology to the 50 US states during the early phase of COVID-19 in 2020. We find that local differences in mobility responses and other parameters imply a wide variation of the economic efficiency of lockdown policies across states. To quantify economic efficiency of lockdown policy (henceforth ``lockdown efficiency") we use mobility as a proxy for economic activity and measure mobility lost if lockdowns would have been used to save the same number of lives as have been saved through information-based voluntary social distancing in a given state. We find that lockdown efficiency varies widely. For example, for US states in the 25th percentile of the lockdown efficiency distribution, lockdowns could have saved the same number of lives as voluntary social distancing, but allowing for 75% more mobility until June 2020. In contrast, for US states in the 75th percentile of the lockdown efficiency distribution, using lockdowns instead of voluntary social distancing would have implied almost 20% less economic activity. In other words, the efficiency of lockdown policies varies substantially across states. To investigate the role of differences in local information-based voluntary social distancing, we impose different mobility responses to reported case and fatality counts. Our information-based voluntary social distancing model suggests that these mobility responses can be systematically influenced by the credibility of local government reports. To provide a reasonable counterfactual for changes in credibility of local government reports, we compare the number of lives saved by uniformly imposing voluntary

social distancing responses in places with low information transmission like West Virginia compared to relatively high information transmission places like Massachusetts. Using West Virginia parameters for the whole US implies over 240,000 additional fatalities before June 2020. In contrast, imposing parameters from Massachusetts across all US states only saves an additional 24,000 lives before June 2020. This shows that the existence of important cost-benefit asymmetries of these information policies, as low-credibility information policies are much more costly in terms of fatalities than high-credibility information policies are beneficial in saving lives. Our quantitative evidence on how local information policies effect pandemic outcomes, to our knowledge unique to this study, highlights the importance of credible information due to voluntary social distancing."

Furthermore, the discussion and conclusions are difficult to follow. I think that they need to be rewritten to better reflect the interesting research results and to highlight the authors' key contributions.

Thank you for pointing this out. In response, we have added are more detailed discussion, highlighting our key contributions. We reprint the key paragraphs here for your convenience:

"We develop a model of information-based voluntary social distancing that is rich enough to quantify the effect of credible local information on case and fatality counts. Our model incorporates the insights highlighted by empirical studies that document, but do not model, the impact of beliefs and the information environment on social distancing ([16], [29], [30]). Our approach extends work by [7], who model social distancing with a compartmental model, by allowing social distancing to be endogenous to the information environment. Our extension is critical to understand what policies or other determinants can influence voluntary social distancing.

This paper also contributes to a small literature of hybrid approaches between Machine Learning and traditional compartmental epidemiological models (henceforth ``hybrid approach"). Some hybrid approaches such as [31] and [32] use very basic compartmental models, which do not allow for voluntary social distancing at all. Based on the literature review of 136 COVID-19 forecasting models in [1], we identified 3 studies that use both a hybrid approach and daily mobility data: [33], [34] and [35]. Our paper extends these papers by also allowing for a feedback from cases and fatalities to mobility through an information channel. To the best of our knowledge, ours is therefore the only forecasting and policy model that utilizes a hybrid approach while allowing for a feedback of local information on cases and fatalities to mobility.

Another hybrid approach we are aware of is pursued by [36], who combine an epidemiology-founded version of Bayes' Law with Machine Learning-based infection-prediction using symptoms to provide a sample-selection corrected measure of the active prevalence of COVID-19. However, their approach does not allow for counterfactual analysis of NPIs, such as lockdown policies."

Thank you again for your guidance and constructive feedback. Your suggestions have helped to considerably strengthen the paper.

Sincerely, The authors
