# Supplementary material for: What drives the effectiveness of social distancing in combating COVID-19 across U.S. states?
Source: PLoS One. 2025 May 12;20(5):e0308244. doi: 10.1371/journal.pone.0308244 (PMC12068638; doi:10.1371/journal.pone.0308244)
Supplement: Attachment — Submitted filename: Referee Responses June 2024.pdf [file pone.0308244.s002.pdf]

1 Reviewer 1: R1

Dear Reviewer,
thank you for the opportunity to revise our work for PLOS-One.
For your convenience we reprint below your original review letter and provide a point-by-point response to your review. We responded to each of your points below in *blue italics* to make it easier for you to read.
Thank you for your thoughtful comments, as they have considerably strengthened the paper.

Paper is well written and can be accepted after the following corrections:
(1) There is overlap in text in Figures 2, 5, 6, ... Authors are suggested to update all these figures and ensure their proper representation.

Thank you for catching this typo, we have fixed these figures.

(2) Caption of Figure 15 is not informative as it does not explain blue and red curves.

Thank you for catching this, we have added in notes to this figure.

(3) Authors are suggested to update the caption of Figure to make them more informative.

Thank you for highlighting this shortcoming. We have fixed the captions to these figures to be more informative.

(4) Reproducibility of the study is important for reliability of the work and also for future research and improvements. Therefore, authors are suggested to provide computer code/programmes developed and used for this study in some publicly open repository like GitHub/ResearchGate or some other open source storage.

Thank you for encouraging reproducibility. We completely agree; our plan is to publish the code on GitHub after the paper is accepted.

(5) Authors are suggested to provide limitations and future directions of the research.

We have added limitations and future directions to the conclusion. Specifically, the conclusion now states:

> *This paper has developed a new methodology to evaluate the effectiveness of social distancing during COVID-19. To achieve this, we combine an extended compartmental model of a pandemic with Machine Learning and a new theory of information-based voluntary social distancing.*
>
> *Qualitatively, the model shows that information-based voluntary social distancing*

was an important feature of the COVID-19 policy response. Quantitatively, our calibrated model suggests the size of this effect may be large enough to be important. Of course, the exact numbers depend on specific calibrations and other model assumptions. The model we provide is tractable enough for future researchers to use and update with different calibrations or model assumptions.

Future research can use our model to analyze at least three additional policy-relevant questions. First, how do different policy alternatives, such as (1) proactive testing and quarantining, (2) increased symptom-based testing, and (3) efforts to increase public attention to published case counts, quantitatively differ in slowing the spread of pandemics like COVID-19? Second, what are the economic implications of these different non-pharmaceutical interventions? Our framework already quantified the reduction in mobility-based economic activities, such as going to work and grocery shopping, but we have not directly translated these mobility changes into unemployment or GDP numbers (although doing so, using regularities such as Okun's Law is straightforward). Third, are there important policy complementarities between different non-pharmaceutical interventions? For example, more aggressive testing might increase case counts, while governments can also influence the degree of voluntary social distancing by focusing the public's attention. Might a combination of these two policies disproportionately slow the spread of the virus down? These questions can be addressed by the framework developed in this paper, and we leave them for future research.

2 Reviewer 2: R2

Dear Reviewer,
thank you for the opportunity to revise our work for PLOS-One.
For your convenience we reprint below your original review letter and provide a point-by-point response to your review. We responded to each of your points below in *blue italics* to make it easier for you to read.
Thank you for your thoughtful comments, as they have considerably strengthened the paper.

Try to improve Figure No. 13, 14, and 16. It is not very clear.

Thank you for highlighting this shortcoming. We have fixed the captions to these figures to be more informative.

And add some good reference in the literature, i. e. 1. Singh, A., & Bajpai, M. K. (2020). SEIHCRD model for COVID-19 spread scenarios, disease predictions and estimates the basic reproduction number, case fatality rate, hospital, and ICU beds requirement. Computer Modeling in Engineering & Sciences, 125(3), 991-1031. 2. Singh, A., Bajpai, M. K., & Gupta, S. L. (2023). A Time-dependent mathematical model for COVID-19 transmission dynamics and analysis of critical and hospitalized cases with bed requirements. In Machine Vision and Augmented Intelligence: Select Proceedings of MAI 2022 (pp. 85-120). Singapore: Springer Nature Singapore. 3. Singh, Avaneesh, Saroj Kumar Chandra, and Manish Kumar Bajpai. "Study of non-pharmacological interventions on COVID-19 spread." Computer Modeling in Engineering & Sciences 125, no. 3 (2020): 966-989. 4. Singh, Avaneesh, and Manish Kumar Bajpai. "A compartmental Mathematical model of COVID-19 intervention scenarios for Mumbai." In Machine Vision and Augmented Intelligence: Select Proceedings of MAI 2022, pp. 121-146. Singapore: Springer Nature Singapore, 2023.

Thank you for highlighting these papers for us.They all use a similar updated SEIR model methodology, and we have added citation and discussion to them in the updated manuscript when discussing the baseline SEIR models.

3 Reviewer 3: R3

Dear Reviewer,
thank you for the opportunity to revise our work for PLOS-One.
For your convenience we reprint below your original review letter and provide a point-by-point response to your review. We responded to each of your points below in *blue italics* to make it easier for you to read.
Thank you for your thoughtful comments.

The article is relevant, very well written and with important results. The authors made the corrections proposed by the other reviewers.

Thank you, we appreciate your comments throughout the process.

4 Reviewer 4: R4

Dear Reviewer,
thank you for the opportunity to revise our work for PLOS-One.
For your convenience we reprint below your original review letter and provide a point-by-point response to your review. We responded to each of your points below in *blue italics* to make it easier for you to read.
Thank you for your thoughtful comments, as they have considerably strengthened the paper.

Thanks for giving me the opportunity to review this manuscript. The retrospective investigation of the effectiveness of social distancing is an important step in preparing for future pandemics. The manuscript introduces various thoughts and deliberation that shed new light on this complex system.
On the other hand, I am afraid that the presented model outcomes are highly uncertain as the model involves a large number of calibration parameters. Moreover, some of the parameter estimates, which are presented as model results, seem to be rather unrealistic. For instance, authors claim that death rates have been fallen within 27 days from 10% to 0.003% due to changes in treatment alone. The infection fatality rate is estimated to 9.15% at the beginning of the outbreak. According to the model this value eventually dropped to 0.003%. Similarly, the basic reproduction seems to be too high (R0=6). Results of other studies significantly deviate from the numbers presented in this study.

Thank you for highlighting these limitations. We have included these limitations to the manuscript to highlight for the reader how the calibrations may be changed to change outcomes. We agree with you that R0 =6 is high. In our model, we estimate a time-varying R0 and the model quickly moves away from R0. We began with R0 = 6 due to the experience in Italy and we show that our calibrated model is robust to this starting point because it adjusts quickly. We also now cut the discussion of the quantitative results for the falling death rates. We instead focus on the qualitative results. Specfically, we state:

> *This death rate should be interpreted as the death rate for symptomatic people since only symptomatic people can die in our model. The model estimates that in the median state, death rates for symptomatic people dropped substantially through the estimation period for the median state. This drop implies an impressive improvement in learning to treat the disease.*

We have also removed the quantitative discussion of IFR.

Moreover, R0 and IFR are highly dependent on the local geography of social networks, culture, habits, age structures, health care, seasonality and many other factors. In addition

to uncertainties in parameter estimates, misperceptions may also originate from the modeling paradigm being used. For instance, the aggregated (equation based) and distributed models (individual or agent based) tend to produce very different results even if the input parameters are the same.

Thank you for highlighting this difference in the literature. We agree, it is important to acknowledge how different models behave in the broader literature and have added this discussion to the manuscript. Specifically, we now state:

> *We note that R_0 and the infected death rate are highly dependent on many factors, including the local geography of social networks, demographics, and culture. We also note that models in the literature have produced different results based on whether they were aggregated (equation-based) or distributed models (e.g., individual or agent-based). These caveats should be considered when interpreting our results.*

In the light of these arguments, I'm in doubt that a model of this type can be used to make quantitative forecasts or retrospective estimates on fatality numbers under given conditions. To deal with that problem I suggest to

Thank you for the roadmap that follows. We have edited the manuscript accordingly. In particular, we (1) put the focus on the qualitative model outcomes, (2) compared our quantitative results with other studies, (3) downplayed quantitative results, especially those from hypothetical conditions, (4) discussed the limitations of the calibration, (5) checked our results for plausibility, and (6) discussed the limitations of the data.

We address your individual points in more detail below, highlighting several places in the paper where we compare our calibration with other studies, consider limitations of these calibrations, and potential extensions for future research.

1) Put a focus on qualitative model outcomes (e.g. voluntary social distancing is more effective than state lockdowns, regional differences and reason why, economic implications etc.). These are the results that should be highlighted in the abstract (e.g. ...results show that effect of voluntary social distancing is more important than the impact of state lockdowns...).

Thank you for this comment. We have cut the quantitative discussion in the abstract and focused on the qualitative model outcomes. Specifically, we have changed the abstract in the following way:

Removed: We find there would have been 240,000 more fatalities by June 2020 if people in all states would have voluntarily socially distanced like people in the least informed US state,

and 25,000 fewer fatalities if people in all states would have responded like people in the most informed state.

Added: We find that voluntary social distancing is higher in more informed states, and increasing information could have substantially changed social distancing and fatalities.

2) If quantitative results are presented, compare with results of other studies (IFR, R0 etc.).

Thank you. We have de-emphasized the quantitative results. In addition, we have connected our results with those in other studies. For example, we say,

> *We start by setting the initial virality R_0 of COVID-19 to 6 based on evidence by (21) on the spread of COVID-19 during the early phases of the outbreak in Wuhan, China. However, it is important to note that the model will estimate time variation in the virality R_t*

In this case, it is important to note that the initial R_0 value is not that important as our model estimates the dynamic R_t and we show that the model is robust to different starting values of R_0.
We also connect the other calibrated numbers to the literature. For example, we say,

> *The second calibrated parameter is the average incubation period, which we set to 5 days, so that $\sigma = 1/5$. This parameter value is consistent with the evidence in (22). The third calibrated parameter is the average resolution time, which we set to 12 days, so that $\theta = 1/12$, based on evidence from (23). Of course, our specific quantitative results depend on these calibrated numbers. Future researchers can use different calibrations based on future studies.*

3) Avoid mentioning quantitative results that cannot be validated (e.g. fatalities under hypothetical conditions) or at least add a statement on the uncertainty associated with such estimates.

Thank you. Now, when we discuss the hypothetical conditions, we add a statement of uncertainty and focus on the qualitative rather than quantitative results. Specifically, we say,

> *The panels in Figure* **??** *show the qualitative results. In these panels, the blue lines correspond to the optimal ensemble model estimates from Figure* **??**. *Of course, there is uncertainty with these hypothetical conditions, and uncertainty is driven both by model assumptions and calibrations. We contrast this estimated and predicted path with two counterfactuals from the model. First, the grey dashed line is the infection, fatality, and mobility time path without state lockdowns but with voluntary social distancing. Second, the black dotted line displays the*

same time paths for a counterfactual without voluntary social distancing but with state lockdowns. As the counterfactual panels show, in both West Virginia and Massachusetts, the effect of voluntary social distancing is more important than the impact of state lockdowns: in general, the dotted lines are above the dashed grey lines.

4) Discuss the limitations and uncertainties of calibration procedures. In theory, calibrated model parameters can be wrong while model outcomes (like fatalities and cases over time) correctly reproduce statistical records.

Thank you for this point. We discuss the uncertainty and use an ensemble model to address model uncertainty. Specifically, we say

> *However, before turning to the results, we want to point out an important caveat. We will present "ensemble averages" of parameters, defined as weighted values of parameters for all models in the optimal ensemble, where weights reflect the optimal ensemble weight. These ensemble averages are meant as an easy way to indicate what models estimate. However, it is unlikely that these estimates themselves would exactly give the ensemble models' estimated paths since all these models are highly non-linear. For the ensemble-averaged parameters, we will also mainly focus on the median values, but the reader should be aware that extreme values that are estimated quantitatively matter in the non-linear models considered here.*

5) Where applicable check your result for plausibility. For instance, other studies showed that the proportion of asymptomatic is dependent on age structures. Are your results consistent with that assumption?

Thank you for pointing this out. We now discuss the model assumptions we use and how future work could update the work for other assumptions on asymptomatic cases. Specifically, we write,

> *Specifically, in the model, the possibility of an asymptomatic infection enters in the stages after the initial exposure. Asymptomatic infections are assumed to have the same transmission rate as symptomatic infections and will have the same duration of infectious and resolving states, but will never result in death. While we are aware that this is potentially a strong assumption, we also point out that it is straightforward to relax it to allow for lower transmission rates by asymptomatic individuals.*

We also discuss our bounds for symptomatic cases,

> *First, we impose an upper bound of death rates for symptomatic people of 15%, consistent with the case fatality rate of 15%, which prevailed in Italy at the height of the COVID-19 crisis in that country. Italy's case fatality ratio, in turn, is the highest currently reported case fatality ratio in the world. Second, we bound the probability of being asymptomatic, conditional on infection, to values* $\alpha \in [0.05, 0.8]$*. The lower bound corresponds to the fraction of patients in (25) who never developed symptoms, while the upper bound corresponds to the upper bound of estimates for* α *in randomized testing data in (26). Most of the existing estimates for* α *comfortably fall within these bounds, such as evidence from the Diamond Princess at* $\alpha = 0.18$ *in (14) and* $\alpha = 0.45$ *in (27).*

6) Address limitations of the data: For instance, Google mobility data is used to quantitatively measure people's response: Wearing face masks or keeping a distance may lead to a decoupling between mobility volumes and infectious contacts. Mobility data alone cannot address such dependencies.

Thank you for highlighting this caveat. We now include the different dependencies in our discussion of mobility and transmission. A nice feature of our model is that we allow the effectiveness of social distancing to vary by location through the parameter ψ*. These differences are meant to capture many of these dependencies, albeit in a reduced-form way. We highlight this by now saying (see bolded addition),*

> *In other words, disease transmission is driven by the way randomly matched people interact with each other, captured by the variable* m_t*, which denotes mobility. Lower mobility* m_t *corresponds to a higher degree of social distancing, which will slow the disease spread. Importantly, we allow the effectiveness of social distancing efforts to vary by location through the parameter* ψ***, which would also capture variances in the efficacy of distancing due to, for example, masking efforts.***

Some remarks concerning the formal appearance:

1) Results are presented in the introduction section (lines 80-110), which is kind of unusual. I am however not quite familiar with the formal criteria that are used by PLOS.

We would be happy to remove the main results from the introduction if asked to do so by the editorial staff. We front loaded the main results there, to save time for many readers, but we would gladly follow formatting criteria used by the journal if it is at odds with this approach.

2) Figure 2 show changes in mobility and unemployment. Mobility reduction is associated with higher unemployment. Does the change in unemployment happen between April 2019

and April 2020? What is meant by year-over-year? Please specify in figure 2. It is also not clear how you use Google Mobility data in Fig. 2. Did you take the average of economically relevant categories such as mobility for work, grocery shopping etc.? Moreover, mobility reduction may have caused unemployment. Alternatively, unemployment may have caused a reduction in workplace mobility. Please explain.

Thank you for highlighting the imprecision of Figure 2. We have updated the figure, the figure notes, and discussion to clarify. Briefly, we used economically relevant categories for mobility.

3) In line 167 you say that Fig. 3 shows relative mobility in June. According to the y-axis of Fig. 3 it is spring 2020. What month or season is shown on the y-axis?

Thank you highlighting this point about Figure 3. We have now updated it. As we explain in the figure notes now, both the y-axis and x-axis are averages. The y-axis is an average of daily data from February to June 2020, while the x-axis is an average of polls until May 2020 by FiveThirtyEight.com.

4) Fig.4 : What time intervals and time span are used? Spring in weekly time steps? Figures are not self-explanatory!

Thank you for highlighting this point in Figure 4. We have now updated it and now state in the note that "Each data point is a different day in April 2020". We have also added figure notes for all other figures and hope they are easier to understand even without reading the main text in which the figures are discussed.

5) The correlations presented in Fig. 6 and 7 seem to be rather weak. Please provide coefficients.

Thank you for highlighting this point about Figures 6 and 7. We have now included the fit trendline equations with standard errors and adjusted R^2 for all the figures. While we agree these fits are worse than the other relationships (i.e. they have the lowest R^2, we note that the coefficients on the regressions are all statistically significant at $< 1\%$).

6) In line 202 it is stated that Fig 8 shows negative correlation. This looks like positive correlation.

Thank you for highlighting this confusing passage. We have updated the writing to clarify.

Specifically, we now state:

> *We define "responsiveness" to case counts as the absolute value of the prevalence elasticity, or $|\mu_1|$. Figures 6, 7, and 8 show that responsiveness to case counts systematically differs across states. The responsiveness decreases with the President's net approval rating and increases with education attainment. Further, and perhaps surprisingly, responsiveness $|\mu_1|$ is positively correlated with initial mobility, Figure 8.*

7) I suggest including full description of abbreviations in Fig. 10, which would significantly improve readability.

Thank you for this suggestion, we have added the definitions of variables and parameters in this flow chart of the model.

8) line 310: In other words. . . .

Thank you for pointing this out. We have corrected this typo.

I am looking forward to seeing your revised manuscript!

Thank you for your constructive and detailed suggestions, they have helped us to considerably strengthen and clarify the paper.

References

[1] Nixon, K., Jindal, S., Parker, F., Reich, N.G., Ghobadi, K., Lee, E.C., Truelove, S. and Gardner, L. An evaluation of prospective COVID-19 modelling studies in the USA: From data to science translation. The Lancet Digital Health, 2022

[2] Murray, C.J. Forecasting COVID-19 impact on hospital bed-days, ICU-days, ventilator-days and deaths by US state in the next 4 months. MedRxiv, 2020

[3] Yglesias, M. The Trump administration's "cubic model" of coronavirus deaths, explained. https://www.vox.com/2020/5/8/21250641/kevin-hassett-cubic-model-smoothing

[4] Kermack W, McKendrick A. A contribution to the mathematical theory of epidemics, part I. Proceedings of the Royal Society of London. 1927

[5] Brauer F, Castillo-Chavez C, Feng Z. Mathematical Models in Epidemiology. Springer New York. 2019

[6] Singh A, Bajpai M.K. SEIHCRD Model for COVID-19 Spread Scenarios, Disease Predictions and Estimates the Basic Reproduction Number, Case Fatality Rate, Hospital, and ICU Beds Requirement. Computer Modeling in Engineering & Sciences, 2020

[7] Philipson T. Economic Epidemiology and Infectious Diseases. Handbook of Health Economics, 2000

[8] Galanis, G., Di Guilmi, C., Bennett, D.L. and Baskozos, G. The effectiveness of Non-pharmaceutical interventions in reducing the COVID-19 contagion in the UK, an observational and modelling study. PLOS One, 2021

[9] Seegert, N., Gaullin, M., Yang, M., Navarro-Sanchez, F. Information Revelation of Decentralized Crisis Management: Evidence from Natural Experiments on Mask Mandates. Working Paper, University of Utah

[10] Chetty R, Friedman J, Hendren N, Stepner M. How Did COVID-19 and Stabilization Policies Affect Spending and Employment? A New Real-Time Economic Tracker Based on Private Sector Data. NBER Working Paper. 2020

[11] Gros C, Valenti R, Schneider L, Valenti K, Gros D. Containment efficiency and control strategies for the Corona pandemic costs. Working Paper, UC Berkeley. 2020

[12] Fernandez-Villaverde, J., Jones, C. Estimating and Simulating a SIRD Model of COVID-19 for Many Countries, States, and Cities. Working Paper, Stanford University. 2020

[13] Berger, D., Herkenhoff, K., Mongey, S. An SEIR Infectious Disease Model with Testing and Conditional Quarantine. NBER Working Paper. 2020.

[14] Mizumoto K, Kagaya K, Zarebski A, Chowell G. Estimating the asymptomatic proportion of coronavirus disease 2019 (COVID-19) cases on board the Diamond Princess cruise ship, Yokohama, Japan, 2020. Eurosurveillance. 2020

[15] Russell T, Hellewell J, Jarvis I, van Zandvoort K, Abbott S, Ratnayake R, et al. Estimating the infection and case fatality ratio for COVID-19 using age-adjusted data from the outbreak on the Diamond Princess cruise ship. Eurosurveillance. 2020

[16] Stock J. Data Gaps and the Policy Response to the Novel Coronavirus. NBER Working Paper. 2020

[17] Allcott, H., Boxell, L., Conway, J., Gentzkow, M., Thaler, M. and Yang, D. Polarization and public health: Partisan differences in social distancing during the coronavirus pandemic. Journal of public economics, 2020

[18] Muth J. Rational Expectations and the Theory of Price Movements. Econometrica. 1961

[19] Henry D. Forecast Failure, Expectations Formation and the Lucas Critique. The Econometrics of Policy Evalution. 2002

[20] Atkeson A. How Deadly Is COVID-19? Understanding The Difficulties With Estimation Of Its Fatality Rate. NBER Working Paper. 2020

[21] Sanche S, Lin Y, Xu C, Romero-Severson E, Hengartner N, Ke R. High Contagiousness and Rapid Spread of Severe Acute Respiratory Syndrome Coronavirus 2. Emerging Infectious Diseases. 2020

[22] Lauer S, Grantz K, Bi Q, Jones F, Zheng Q, Meredith H, et al. The Incubation Period of Coronavirus Disease 2019 (COVID-19) From Publicly Reported Confirmed Cases: Estimation and Application. Annals of Internal Medicine. 2020

[23] O WH. Report of the WHO-China Joint Mission on Coronavirus Disease 2019 (COVID-19). https://wwwwhoint/docs/default-source/coronaviruse/who-china-joint-mission-on-covid-19-final-reportpdf. 2020

[24] Gouriéroux C, Monfort A. Simulation-Based Econometric Methods. OUP/CORE Lecture Series, Oxford University Press. 1997

[25] Arons M, Hatfield K, Reddy S, Kimball A, James A, Jacobs J, et al. Presymptomatic SARS-CoV-2 Infections and Transmission in a Skilled Nursing Facility. New England Journal of Medicine. 2020

[26] Samore, M., Looney, A., Orleans, B., Greene,T, Seegert, N., Delgado,J., Presson, A., Zhang, C., Ying, Y., Zhang, Y., Shen, J., Slev, P., Gaulin, M., Yang, M., Pavia, A., Alder, S. Seroprevalence of SARS-CoV-2–Specific Antibodies Among Central-Utah Residents. Emerging Infectious Diseases. 2021

[27] Oran D, Topol E. Prevalence of Asymptomatic SARS-CoV-2 Infection. Annals of Internal Medicine. 2020

[28] Hastie T, Tibshirani R, Friedman J. The Elements of Statistical Learning. 2020;Springer: Science+Business Media.

[29] Goolsbee A, Syverson C. Fear, Lockdown, and Diversion: Comparing Drivers of Pandemic Economic Decline 2020. NBER Working Paper. 2020

[30] Brzezinski, A., Kecht, V., Van Dijcke, D. and Wright, A.L. Science skepticism reduced compliance with COVID-19 shelter-in-place policies in the United States. Nature Human Behaviour. 2021

[31] Simonov A, Sacher S, Dubé J, Biswas S. The Persuasive Effect of Fox News: Noncompliance with Social Distancing During the COVID-19 Pandemic Marketing Science, 2021

[32] Rahmadani, F. and Lee, H. Hybrid deep learning-based epidemic prediction framework of COVID-19: South Korea case Applied Sciences, 2020

[33] Delli Compagni, R., Cheng, Z., Russo, S. and Van Boeckel, T.P. A hybrid Neural Network-SEIR model for forecasting intensive care occupancy in Switzerland during COVID-19 epidemics PLOS One, 2022

[34] Arik SO, Li C-L, Yoon J, Sinha R, Epshteyn A, Le LT, Menton V, Singh S, Zhang L, Nikoltchev M, Sonthalia Y, Nakhost H, Kanal E, Pfister T. Interpretable Sequence Learning for COVID-19 Forecasting Advances in Neural Information Processing Systems, 2020 https://arxiv.org/abs/2008.00646v2

[35] Wang L, Wang G, Gao L, Li X, Yu S, Kim M, Wang Y, Zhiling G Spatiotemporal Dynamics, Nowcasting and Forecasting of COVID-19 in the United States https://arxiv.org/abs/2004.14103v4

[36] Bhouri MA, Costabal FS, Wang H, Linka K, Peirlinck M, Kuhl E, Perdikaris, P. COVID-19 dynamics across the US: A deep learning study of human mobility and social behavior Computer Methods in Applied Mechanics and Engineering, 2021

[37] Yang, M., Bertanha, M., Gaulin, M., Seegert, N., Looney, A., Orleans, B., Pavia, A., Stratford, K., Samore, M., Alder, S. What is the Active Prevalence of COVID-19? Review of Economics and Statistics. 2023.
